# Altered white matter architecture in patients with isolated congenital anosmia

**DOI:** 10.1007/s00429-025-02942-4

**Published:** 2025-05-31

**Authors:** Yun-Ting Chao, Yin-Chun Liao, Pei-Shan Hsu, Divesh Thaploo

**Affiliations:** 1 Division of Rhinology, Department of Otorhinolaryngology-Head and Neck Surgery, Taipei Veterans General Hospital, No.201, Sec. 2, Shipai Rd., Beitou District, Taipei, 11217 Taiwan; 2 Department of Otolaryngology, National Yang Ming Chiao Tung University, Taipei, Taiwan; 3 Department of Chinese Medicine, Taipei Tzu Chi Hospital, Taipei, Taiwan; 4 Department of Otorhinolaryngology, Smell and Taste Clinic, TU Dresden, Dresden, Germany

**Keywords:** Congenital anosmia, Functional magnetic resonance imaging, Diffusion tensor imaging, Tract-based Spatial statistics, Fractional anisotropy

## Abstract

Isolated Congenital Anosmia (ICA) is a rare condition characterized by the absence of the sense of smell due to the underdevelopment of the olfactory bulbs and a corresponding lifelong lack of olfactory input to the brain. Previous studies have reported that ICA patients exhibit structural and volumetric alterations in both gray and white matter regions as well as slightly elevated fractional anisotropy (FA) in the orbitofrontal cortex. This study expanded on these findings by utilizing whole-brain diffusion tensor imaging (DTI) with a 3T MR scanner to investigate microstructural changes in the white matter of 8 ICA patients and 14 age- and gender-matched healthy controls (HCs). Tract-based spatial statistics revealed significantly lower FA values in the right superior corona radiata (SCR) of ICA patients compared to HCs (*p* = 0.001), indicating compromised white matter integrity in this region. Elevated mean diffusivity and radial diffusivity values in the right SCR (*p* = 0.009 and *p* = 0.002, respectively) suggest underlying demyelination. In the HC group, FA values in the SCR cluster were positively correlated with odor identification scores (rho = 0.59, *p* = 0.026), highlighting the functional relevance of SCR in processing olfactory information and semantic formation of odor perception. These findings underscore the critical role of early sensory input in shaping the architecture of white matter, and illustrate the impact of congenital olfactory deprivation on the structural integrity and functional organization of neural pathways.

## Introduction

The human olfactory system plays key roles in sensory perception, emotional responses, and memory (Lemogne et al. 2015). Olfactory loss, or anosmia, refers to a significant reduction in the sense of smell, negatively impacting daily function (Whitcroft et al. 2023; Landis et al. 2004). This condition, affecting roughly 5% of the population, can lead to emotional distress and a decrease in one’s quality of life (Croy et al. 2014).

A rarer and more distinct form of olfactory impairment is congenital anosmia, characterized by an inability to perceive odors since birth. This condition can occur in isolation or as part of a broader syndrome, such as Kallmann’s syndrome (associated with hypogonadotropic hypogonadism). Isolated congenital anosmia (ICA), affecting an estimated 1 in 5,000 to 10,000 individuals, is typically associated with the absence or underdevelopment of the olfactory bulbs (Saw et al. 2022). Unlike acquired anosmia, ICA deprives the brain of olfactory input throughout life, offering a unique opportunity to study the impact of lifelong sensory deprivation on neural development and plasticity.

A lack of olfactory input affects the morphology of the brain, with different mechanisms of impairment—such as congenital versus acquired anosmia—resulting in distinct patterns of neuroplasticity (Postma et al. 2021; Manan et al. 2022). In ICA, where olfactory input is absent from birth, increased cortical thickness is observed in brain areas devoted to olfactory processing, such as medial orbitofrontal cortex (OFC). This phenomenon has been attributed to a lack of synaptic pruning of redundant neural connections during postnatal development (Peter et al. 2020; Frasnelli et al. 2013). In acquired anosmia, where olfactory input is lost after normal development, the same regions exhibit volume reduction and decreased cortical thickness (Han et al. 2018; Yao et al. 2018). These contrasting patterns underscore the critical role of sensory input in shaping the brain architecture during the developmental and adult stages of life.

Volumetric analysis has also revealed alterations in the white matter architecture of ICA patients, particularly in the superior longitudinal fasciculus, which is located mediodorsal to the insula and dorsal to the parietal operculum (Frasnelli et al. 2013). This alteration may be attributed to a lack of synaptic pruning in the absence of olfactory input during early life. However, focal volumetric differences in white matter do not fully capture architectural integrity, particularly when dealing with axonal myelination. Considering the importance of white matter as a conduit for neural connectivity, assessing its functional and structural integrity requires methods that go beyond volumetric analysis.

Diffusion tensor imaging (DTI) is a robust tool for assessing the structural integrity and connectivity of white matter. This technique estimates water diffusion properties within structured spaces, such as axons, where diffusion is constrained along certain directions but remains unconstrained in others. A key metric derived from DTI is fractional anisotropy (FA), which quantifies the degree of coherence in directional water diffusion within a given area (Alba-Ferrara and de Erausquin 2013). FA is considered a marker of axonal myelination, providing valuable insight into the microstructure of white matter tracts under various neurological conditions, including the aging process (Kochunov et al. 2011), neurodegenerative disease (Owens-Walton et al. 2024), schizophrenia (Alba-Ferrara and de Erausquin 2013), and traumatic brain injury (Wallace et al. 2018). Researchers have recently reported elevated FA values in the OFC of ICA patients (Thaploo et al. 2022), consistent with increased gray matter thickness in the same region (Frasnelli et al. 2013). This suggests the possibility of alterations in axonal myelination in individuals with ICA, underscoring the need for further analysis of white matter tract changes using advanced DTI techniques.

Other diffusion parameters commonly derived from DTI include mean diffusivity (MD), axial diffusivity (AD), and radial diffusivity (RD). MD indicates the average distance that water molecules diffuse across three orthogonal axes, regardless of directionality. In healthy individuals, MD values provide insight into the density and structural stability of cellular structures (Vos et al. 2012; Beaulieu 2002; Takeuchi and Kawashima 2018). Damaged white matter typically presents low FA and high MD values (Owens-Walton et al. 2024).

AD quantifies water diffusion along the longitudinal axis of axons, serving as an indicator of axonal integrity. RD quantifies water diffusion perpendicular to axonal fibers, serving as an indicator of myelin integrity. In pathological conditions, elevated AD values are typically associated with reduced axonal density or caliber, whereas elevated RD values typically indicate demyelination linked to chronic disease (Kumar et al. 2012).

Understanding how the olfactory system processes sensory information requires insight into the connectivity architecture of the brain and its potential reorganization in the absence of early-life olfactory input. The extent to which olfactory inputs influence brain connectivity has yet to be fully elucidated. In the current study, we investigated the impact of ICA on structural connectivity along white matter pathways. Tract-based Spatial Statistics (TBSS), a whole brain white matter skeleton-based approach, was used to compare DTI metrics from ICA patients and age- and gender-matched healthy control subjects. This enabled the identification of fine-grained structural differences between the groups, shedding light on the role of olfactory input in the development and organization of white matter tracts.

## Methods

### Participants

This study recruited patients diagnosed with ICA at Taipei Veterans General Hospital, based on the following inclusion criteria: (1) a lifelong absence of olfactory perception since birth, (2) a diagnosis of anosmia confirmed through olfactory psychophysical testing using the Sniffin’ Sticks Test (Oleszkiewicz et al. 2019), and (3) imaging evidence demonstrating hypoplasia or agenesis of the olfactory bulbs.

Age- and gender-matched healthy participants with normal olfactory function, as confirmed using the Sniffin’ Sticks Test, were included as healthy controls (HC). Exclusion criteria for both ICA patients and HC participants included the following: (1) the presence of brain or nasal lesions, such as stroke or tumors; (2) a history of head trauma resulting in loss of consciousness, intracranial hemorrhage, or craniotomy; and (3) psychiatric disorders as defined by DSM-IV Axis I or II, such as schizophrenia or major depressive disorder. As congenital anosmia is a rare condition, the sample size in this study was determined by the availability of patients. Healthy controls were recruited accordingly to match the patient group. Thus, a priori power analysis was not performed.

The study procedures adhered to the Declaration of Helsinki and received approval from the local ethics committee of Taipei Veterans General Hospital (2022-05-004BCF). All participants provided written informed consent prior to enrollment.

### Olfactory function test

All participants underwent a comprehensive otolaryngological examination to rule out intranasal pathologies and completed psychophysical testing to assess olfactory function. The Sniffin’ Sticks Test (Burghart Messtechnik GmbH, Holm, Germany) was used to evaluate three key components of olfactory performance: odor detection threshold (T), discrimination (D), and identification (I) (Hummel et al. 1997; Oleszkiewicz et al. 2019).

The threshold (T) test was used to determine the olfactory threshold for n-butanol based on an adaptive staircase procedure with a three-alternative forced-choice presentation. This involved presenting participants with three pens — one containing n-butanol and two containing an odorless solvent — and having them identify the pen with the odor. The discrimination (D) test involved the presentation of 16 pen triplets in which two contained the same odor and the third contained an odd odor. The pens were presented in random order, and participants were required to identify the odd one out. The identification (I) test involved identifying 16 odors common to Taiwanese people (Shu et al. 2007) from a list of four descriptors provided for each pen.

The combined score from the three subtests constituted the TDI score, which served as a comprehensive measure of olfactory performance. Normosmia was defined as a TDI score exceeding 30.5, while anosmia was defined as a TDI score of 16 or below (Hummel et al. 2007).

### Neuropsychological tests

Considering the correlation between anosmia and the development of neuropsychological conditions (e.g., depression), all participants were required to undergo assessments using the Beck Depression Inventory-II (BDI-II) (Beck 1996), ensuring a comprehensive understanding of potential psychological influences on white matter architecture via neuroplasticity.

### Image acquisition

All participants underwent the same imaging protocol involving whole-brain diffusion-weighted scanning at National Yang Ming Chiao Tung University. Scanning was performed using a 3.0T MRI machine (MAGNETOM Trio™, Siemens, Erlangen, Germany) equipped with a 12-channel head coil under the head-coil gradient configuration. Diffusion-weighted images were obtained using generalized auto-calibrating partial parallel acquisition (GRAPPA) sequencing technology with the following parameters: repetition time (TR; 7900 ms), echo time (TE; 79 ms), slices (70 with thickness of 2 mm and no interslice gaps), voxel size (2 × 2 × 2 mm^3^), field of view (256 × 256 mm^2^), matrix size (128 × 128), echo spacing (0.85 ms), acceleration factor (4), number of diffusion directions (64), *b*-value (900 s/mm² plus a single b-value of 0 s/mm^2^ image), and EPI factor (128), and an anterior-to-posterior (AP) phase-encoding direction.

Participants also underwent high-resolution T1-weighted structural scanning using a magnetization-prepared rapid acquisition gradient echo (MPRAGE) sequence, with the following imaging parameters: TR (2530 ms), TE (3.03 ms), flip angle (7 degrees), voxel size (1 × 1 × 1 mm^3^), slice thickness (1 mm), field of view (224 × 256 mm^2^), matrix size (224 × 256), and slices (192).

### Image preprocessing

DTI images were prepared and analyzed using the FMRIB Software Library (FSL, version v6.0.7; https://fsl.fmrib.ox.ac.uk/fsl) (Jenkinson et al. 2012), utilizing the FSL Diffusion Toolbox (FDT). Preprocessing began with the removal of data unrelated to brain tissue using the Brain Extraction Tool (BET), followed by the generation of a binary brain mask for each subject based on their individual average (Smith 2002). Note that we did not perform Gibbs ringing correction, as it is not included in the FSL pipeline and is typically applied in studies using high-resolution or multi-shell diffusion data (Mazur-Rosmus and Krzyżak 2024). Eddy current correction was then applied to mitigate head motion artifacts and eddy current distortion. Quality control was conducted using QUAD (Quality Assessment of Diffusion), the exclusion criteria of which were head motion with an absolute displacement exceeding 2 mm, the voxel size, or a relative displacement exceeding 0.3 mm (Bastiani et al. 2019). Next, the DTIFIT tool was used to compute diffusion tensors and generate parameter maps of white matter, including FA, MD, AD, and RD. Note that FA quantifies the difference between the largest eigenvalue and the other two, providing an indication of the white matter microstructure. MD is the average of all three eigenvalues, and is sensitive to variations in cellularity, edema, and necrosis. AD focuses solely on the first eigenvalue, which is often altered in cases of white matter pathology. RD is the mean of the second and third eigenvalues, with elevated values indicating the possibility of demyelination in white matter (Tromp 2016).

Voxel-wise statistical analysis of diffusion data was performed using Tract-based Spatial Statistics (TBSS) (Smith et al. 2006), implemented in FSL. FA images of all subjects were nonlinearly aligned to the FMRIB58_FA template in the Montreal Neurological Institute 152 (MNI152) space, generating a study-specific mean FA atlas to ensure spatial comparability across participants. A skeletonized mean FA image was generated by thinning all aligned FA images with a threshold of > 0.2, thereby excluding voxels corresponding to adjacent gray matter or cerebrospinal fluid. The FA map of each subject was projected onto the FA skeleton using the highest FA values detected in the direction surrounding the tract. MD, AD and RD values were evaluated using the same procedure as FA analysis.

### Statistical analysis

Non-parametric analysis was employed to account for the small sample size and the deviation of some behavioral data from the normal distribution. Between-group comparisons of demographic information and psychophysical measurements were conducted using the Mann-Whitney *U* test for continuous variables and Fisher’s exact test for categorical variables (e.g., gender). Statistical analysis was performed using SPSS software (version 29; IBM, Corp., Armonk, NY, USA).

Brain image analysis was based on voxel-wise between-group comparisons using the FSL Randomize tool (version 2.1). Non-parametric permutation testing was performed to compare FA values across groups. Threshold-free cluster enhancement (TFCE) was used to enhance cluster detection without the need for an arbitrary threshold, and family-wise error (FWE) correction was applied to control for multiple comparisons. Statistical significance was defined as an FWE-corrected *p* < 0.05, with a minimum cluster size of 30 voxels (Winkler et al. 2014).

Significant white matter clusters were identified using the Johns Hopkins University–International Consortium for Brain Mapping white matter atlas (JHU-ICBM-DTI-81, https://neurovault.org/images/1401), serving as a region of interest (ROI) for further analysis. The average signal intensity of FA, MD, AD, and RD within the ROIs were extracted to analyze between-group differences using the Mann-Whitney U test. To control for type I error arising from multiple comparisons across the four diffusion metrics (FA, MD, AD, and RD), Bonferroni correction was applied. Accordingly, the corrected significance threshold was set at *p* < 0.0125 (0.05/4).

Spearman correlation analysis was conducted to examine the relationships between diffusion values within the ROIs and the scores of the Sniffin’ Sticks test in the HC group. We did not conduct a similar correlation analysis on the ICA group due to the nature of the olfactory test, which is based on a forced choice paradigm. Anomic patients may obtain correct answers by chance, rendering their scores devoid of meaningful interpretation (Whitcroft et al. 2017). The level of significance was set at *p* < 0.05.

## Results

### Clinical characteristics of participants

**Fig. 1:**
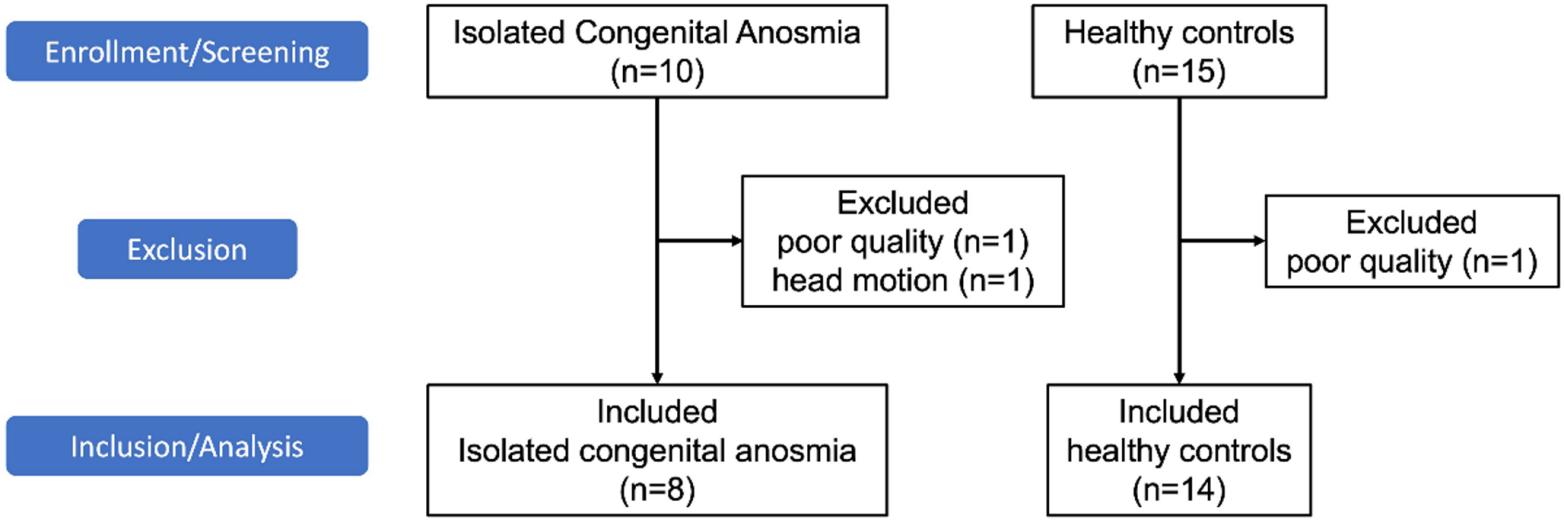
Flowchart illustrating the recruitment and assessment of participants: A total of 25 participants were initially recruited, including 10 patients with isolated congenital anosmia and 15 healthy controls. Three individuals were excluded due to poor scan quality resulting from excessive head motion (> 2 mm). This left 22 individuals (14 healthy controls and 8 patients) for inclusion in the study

A total of 25 participants were initially recruited, 3 of whom were excluded due to poor image quality and excessive head motion (> 2 mm). The final cohort consisted of 22 participants, including 8 ICA patients and 14 age-matched HCs (Fig. 1). The median of age in both groups was 30 years, ranging from 22 to 41 years in the ICA group and from 23 to 42 years in the HC group. The ICA group included 5 males and 3 females, whereas the HC group included 9 males and 5 females.

Olfactory function scores in the ICA group were significantly lower than those in the HC group, which aligned with the inclusion criteria. No significant differences in neuropsychiatric status were observed between ICA patients and HCs, as evidenced by comparable BDI-II scores (Table 1).

**Table 1:** Demographics and clinical characteristics of study cohort

	ICA (*n* = 8)	HC (*n* = 14)	U value	*p* value
Age	30 [22, 46]	30 [23, 43]	54.5	0.92
Gender (M/F)	5/3	9/5	-	†1.00
T (Threshold)	1 [1, 1.25]	7 [4.5, 9.5]	0	< 0.001*
D (Discrimination)	4 [3, 7]	13 [11, 16]	0	< 0.001*
I (Identification)	5 [3, 10]	15 [13, 16]	0	< 0.001*
TDI	10 [7, 16.25]	34.4 [31.75, 40.5]	0	< 0.001*
BDI-II	2.5 [0, 5]	1.5 [0, 14]	47.0	0.53

Data are presented as median [min, max]. Fisher’s exact test† for gender and Mann-Whitney *U* test for the other parameters. Abbreviations: ICA, isolated congenital anosmia; HC, healthy controls; M, male; F, female; BDI, Beck’s Depression Inventory. *Statistical significance

### Group differences in tract diffusion measurements

**Fig. 2:**
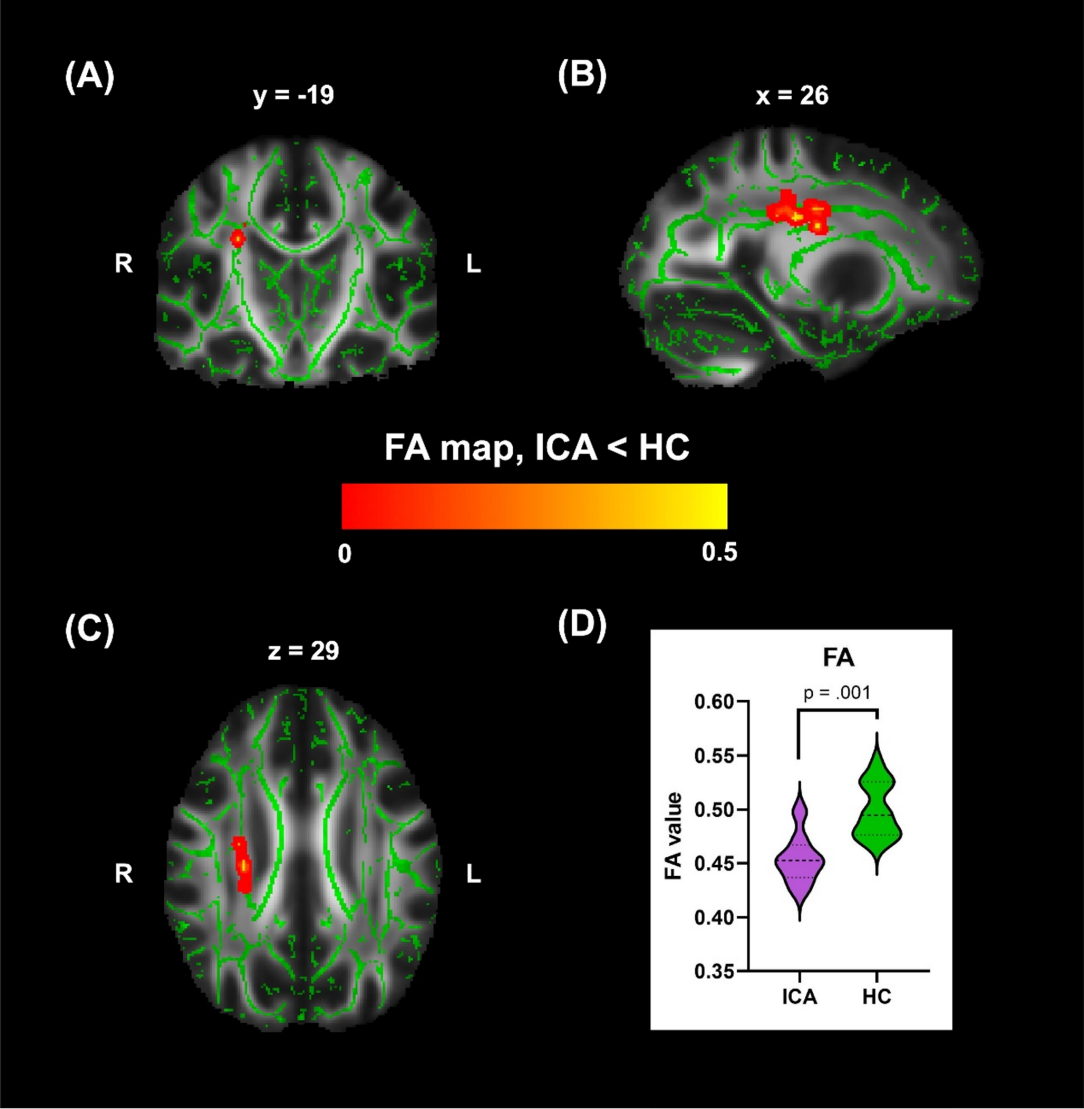
Between-group differences in FA: The highlighted region across (**A**) coronal, (**B**) sagittal and (**C**) axial planes indicates regions in which FA values were lower in the ICA group than in the HC group (ICA < HC). This group difference was observed in the right SCR; (**D**) Violin plot of FA values extracted from the significant region for each group, indicating compromised integrity in the white matter of the right SCR in the ICA group. Abbreviations: FA, fractional anisotropy; ICA, isolated congenital anosmia; HC, healthy controls; R, right; L, left; SCR, superior corona radiata

Whole-brain TBSS analysis at the pre-defined threshold revealed two confluent clusters with significantly low FA values in the ICA group, both located within the right superior corona radiata (SCR) (TFCE/FWE-corrected *p* < 0.05) (Table 2; Fig. 2). No significant clusters were detected for the other diffusion metrics (MD, AD or RD).

**Table 2:** Between-group differences in TBSS analysis of FA data

Cluster location	x	y	z	Cluster size (voxel)
Superior Corona Radiata	25	-21	30	47
	25	-13	34	34

All differences between groups were significant at TFCE/FWE-corrected *p* < 0.05 with voxel cluster size of > 30. Abbreviations: TBSS, tract-based spatial statistics; FA, fractional anisotropy

The Mann-Whitney *U* test was used to compare group differences in the mean FA values extracted from the SCR clusters. The analysis confirmed that FA values in the ICA group were significantly lower than those in the HC group (*U* = 9, *p* = 0.001, Fig. 2D).

**Fig. 3:**
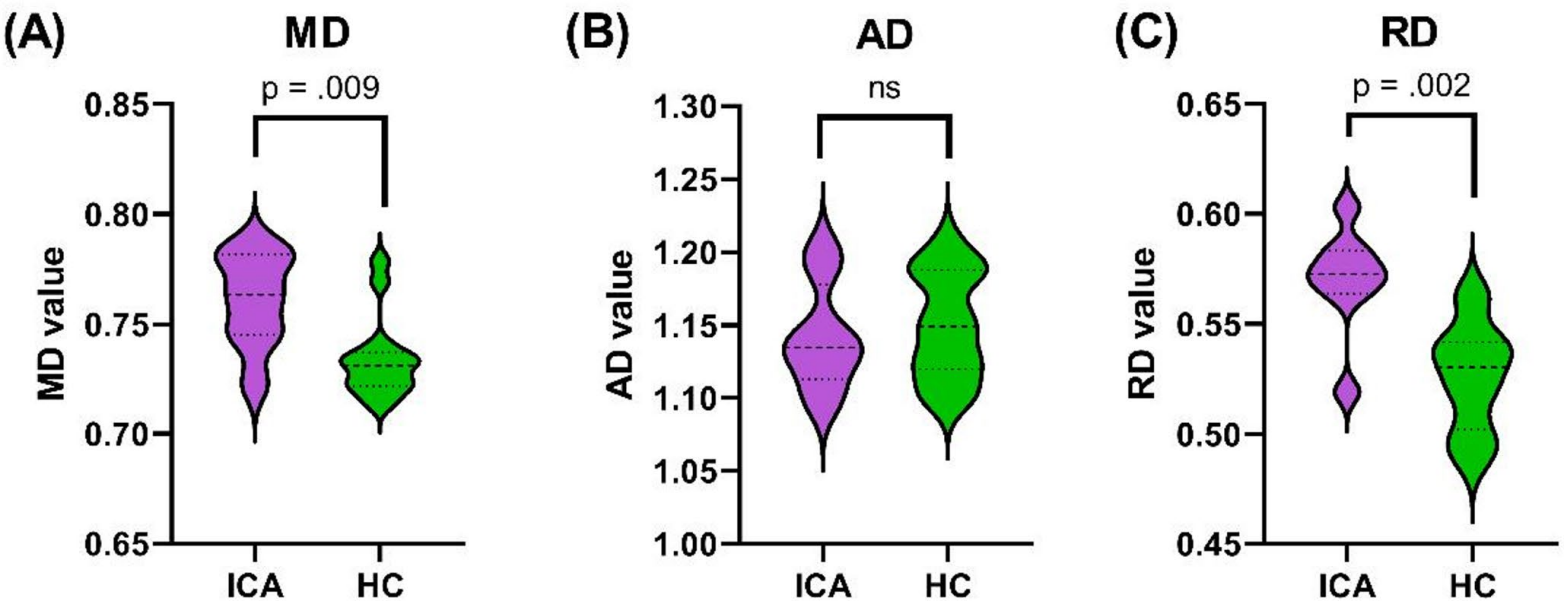
Between-group differences in diffusivity metrics in the right SCR: (**A**) MD; (**B**) AD; and (**C**) RD values (x10^− 3^ mm^2^/sec). The MD, AD, and RD values were extracted from the SCR cluster identified during FA analysis. ICA patients presented higher MD (*p* = 0.009) and RD (*p* = 0.002) values compared to HC. No significant between-group difference was observed in AD values. Abbreviations: ICA, isolated congenital anosmia; HC, healthy controls; AD, axial diffusivity; MD, mean diffusivity; RD, radial diffusivity; SCR, superior corona radiata. Significance was defined as *p* = 0.0125. (0.05/4, the number of DTI metrics examined = 4)

As for the other diffusion metrics extracted from the same SCR clusters, the ICA patients exhibited higher MD (*U* = 18, *p* = 0.009) and RD (*U* = 10, *p* = 0.002) than did the HC group (Fig. 3). These results met the Bonferroni-corrected significance threshold, which was set at *p* = 0.0125 (0.05/4). No significant between-group differences were observed for the AD values (*U* = 50, *p* = 0.68).

### Correlation analysis

**Fig. 4:**
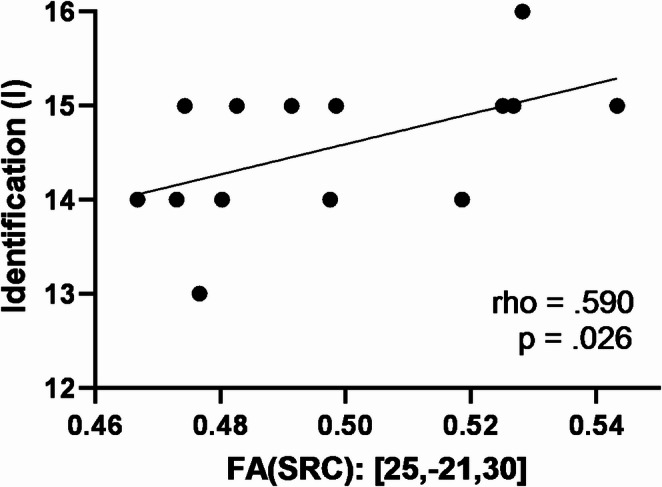
Correlation between FA values in right SCR and odor identification in the HC group. FA values were extracted from the SCR cluster identified during between-group comparisons. Abbreviations: FA, fractional anisotropy; SCR, superior corona radiata; HC, healthy controls

In the HC group, a positive correlation was observed between the mean FA values within the right SCR cluster and odor identification performance (rho = 0.59, *p* = 0.026) (Fig. 4). No significant correlations were identified between MD, AD, or RD values and performance on the threshold or discrimination subtests, or the composite TDI scores.

## Discussion

In the current study, whole-brain DTI metrics were used to investigate alterations in white matter architecture in individuals with ICA. Our findings revealed decreased FA values in the right SCR of ICA patients, suggesting impaired white matter integrity in this region. We also detected significantly elevated RD and MD values in the same region. No significant differences in AD values were detected between the groups. Taken together, these findings suggest that myelin integrity in the right SCR is impaired in individuals with ICA.

Low FA values indicate reduced diffusion directionality at the voxel level, potentially reflecting the compromised microstructural integrity of white matter tracts. Changes such as these can be attributed to alterations in axonal density, myelination, or fiber coherence within the affected tracts (Hermoye et al. 2006). These structural impairments could conceivably disrupt connectivity and functional integration across brain regions (Basser and Jones 2002), contributing to the neurological characteristics observed in individuals with ICA.

One previous study reported elevated FA values in the OFC of ICA individuals (Thaploo et al. 2022); however, we detected a decrease in FA values in the right SCR. Note that the previous study employed a hypothesis-driven approach, focusing on diffusion changes in olfaction processing regions of the gray matter, such as the OFC. In contrast, our study adopted a whole-brain exploratory design, uncovering alterations in major white matter tracts. These findings provide novel insights into central neuroplasticity and structural reorganization associated with congenital olfactory deprivation, highlighting the broader impact of lifelong sensory loss on brain architecture.

Although changes in FA values can be used to localize alterations in the axonal structure of white matter, they do not reveal the mechanisms driving these changes. However, the elevated MD and RD values observed in the right SCR provide additional context, suggesting potential microstructural disruptions such as demyelination. MD, representing average diffusivity within a voxel, is known to increase in cases of tissue damage, edema, or a loss of cellular integrity (Vos et al. 2012; Nakagawa et al. 2023). The increase in MD observed in this study likely reflects a decrease in diffusion barriers within white matter tracts, suggesting impaired structural organization. RD, as the mean of the second and third eigenvalues, is particularly sensitive to changes in axonal myelination, axonal diameter, and axonal packing density (Kumar et al. 2012; Alexander et al. 2007; Song et al. 2002). The absence of a significant difference in AD values indicates that deviations in axonal diameter and density are less prominent in this region (Alexander et al. 2007; Kumar et al. 2012). Collectively, these findings suggest that the primary pathology in the right SCR of ICA patients was associated with demyelination rather than axonal disruption. These changes may be attributed to the long-term effects of congenital olfactory deprivation on the structure of white matter, contributing to altered neural connectivity in the affected areas. One recent study reported that among COVID-19 patients, those with olfactory dysfunction presented higher MD and RD values around the anterior corona radiata, while no intergroup differences in AD were detected (Campabadal et al. 2023). This supports the hypothesis that axonal demyelination in the white matter structure of the corona radiata may be involved in acquired loss of smell.

The SCR carries fibers from the pyramidal tract that converge into the internal capsule, forming a critical pathway for transmitting motor and sensory signals between the cerebral cortex and the brainstem/spinal cord (Jellison et al. 2004). In normal subjects, higher FA values in the SCR are associated with enhanced cognitive abilities (Moeller et al. 2015), reflecting more efficient processing in the recognition and identification of odors. Likewise, our findings in the HC group revealed a positive correlation between FA values in the SCR and olfactory identification scores, which suggests that the integrity of white matter fibers in the SCR corresponds to better olfactory identification ability. Our observation of lower FA values among ICA patients suggests diminished white matter integrity, underscoring a close relationship between impaired connectivity and olfactory dysfunction. Previous studies in Parkinson’s hyposmia have demonstrated connections between the SCR and olfactory areas, such as the OFC and anterior entorhinal cortex. They also reported that impaired functional connectivity between these regions is closely related to reduced olfactory function, highlighting the importance of the SCR in olfactory perception and the profound impact of structural alterations in this region. (Wang et al. 2022).

Alterations in axonal microstructure can also be attributed to a lack of sensory input during early development, extending beyond olfaction alone (Pare et al. 2023). For example, patients with blindness from an early age exhibit reduced FA values and increased MD and RD values in white matter tracts associated with visual as well as non-visual functions (Anurova et al. 2019; Park et al. 2007), presumably due to a disruption in normal myelination. Prelingual deafness has also been linked to low FA values in multiple white matter tracts (Park et al. 2018), attributed to diminished neuronal activity resulting from sensory deprivation. Reduced activity can impede the survival of newly divided progenitor cells, ultimately leading to demyelination (Dietz et al. 2016). In mouse models, early olfactory deprivation through naris occlusion has been shown to induce changes in myelination in olfactory tracts (Collins et al. 2018). Collectively, these findings highlight the critical role of early sensory input in maintaining white matter integrity. They also suggest that congenital sensory loss can induce structural reorganization in related neural pathways.

In this study, BDI-II scores were collected from all participants for descriptive purposes only, and no exclusion criteria were applied based on these scores. The primary rationale for collecting BDI-II data was to ensure that ICA participants did not exhibit elevated depressive symptoms that might confound the neuroimaging results. Notably, the HC group showed greater variability in BDI-II scores, primarily due to one participant reporting mild mood disturbance (score of 14) related to a recent stressful event. However, statistical analysis revealed no significant difference in BDI-II scores between the ICA and HC groups. Therefore, we did not include BDI-II scores as covariates in the primary analyses.

The primary limitation of this study was its small sample size, due to the rarity of ICA and the inherent difficulty in recruiting patients, which may reduce the statistical power and undermine the generalizability of our findings. Nonetheless, we used an age- and sex-matched HC group to reduce confounding variability. Moreover, the observed white matter changes in the SCR are consistent with prior findings in patients with acquired olfactory dysfunction, supporting the plausibility of our results. To address concerns regarding statistical power, we conducted a post hoc power analysis based on the FA values. The effect size (Cohen’s d) was 1.89, indicating a very large effect, and the statistical power was estimated at 98.2%. These results suggest that, despite the small sample size, our study was sufficiently powered to detect significant group differences in FA. Larger cohort studies will be required to verify our findings and further elucidate the impact of congenital olfactory deprivation on the white matter architecture.

Another limitation is the cross-sectional design, which precludes any conclusions pertaining to the progression of white matter alterations over time. Longitudinal studies tracking changes in diffusion metrics from early development through adulthood would no doubt provide a more complete understanding of the dynamics of congenital sensory deprivation. The reliance of this study on structural imaging alone further limited our ability to explore functional implications. Integrating functional imaging studies with structural analysis could provide a more comprehensive picture of how altered white matter integrity affects neural connectivity and olfactory function. Finally, the scope of future research could be expanded by investigating the effects of congenital sensory deprivation across sensory modalities, providing a broader perspective on neuroplasticity and white matter reorganization.

## Conclusions

This study provides valuable insights into the structural alterations of white matter in ICA patients, based on diffusion tensor imaging metrics. The observed decrease in FA values and increases in MD and RD values in the right SCR can be attributed to disruptions in structural organization and potential demyelination associated with congenital olfactory deprivation. These alterations underscore the critical role of sensory input in maintaining white matter integrity and the profound impact that sensory deprivation can have on neural connectivity. Longitudinal and functional imaging studies will be required to further elucidate the relationships among structural changes, neural connectivity, and functional outcomes, providing a comprehensive understanding of the mechanisms underlying neuroplasticity under sensory deprivation conditions.

## Data Availability

The datasets generated and/or analyzed during this study are not publicly available to protect individual privacy. However, they can be obtained from the corresponding author upon reasonable request.
